# Intrapelvic Migration of the Lag Screw in Intramedullary Nailing

**DOI:** 10.1155/2014/519045

**Published:** 2014-12-29

**Authors:** Tomoya Takasago, Tomohiro Goto, Shunichi Toki, Daisuke Hamada, Shinji Yoshioka, Ichiro Tonogai, Takahiko Tsutsui, Yasuaki Tamaki, Keizo Wada, Koichi Sairyo

**Affiliations:** Department of Orthopedics, Institute of Health Biosciences, The University of Tokushima Graduate School, 3-18-15 Kuramoto, Tokushima 770-8503, Japan

## Abstract

Internal fixation with intramedullary devices has gained popularity for the treatment of intertrochanteric femoral fractures, which are common injuries in the elderly. The most common complications are lag screw cut out from the femoral head and femoral fracture at the distal tip of the nail. We report here a rare complication of postoperative lag screw migration into the pelvis with no trauma. The patient was subsequently treated with lag screw removal and revision surgery with total hip arthroplasty. This case demonstrated that optimal fracture reduction and positioning of the lag screw are the most important surgical steps for decreasing the risk of medial migration of the lag screw. Furthermore, to prevent complications, careful attention should be paid to subsequent steps such as precise insertion of the set screw.

## 1. Introduction

Intertrochanteric femoral fractures are common injuries in the elderly. An estimated 1.66 million hip fractures occurred worldwide in 1990, and this number is expected to increase to 6.26 million by 2050 [1]. Management options for these fractures include extramedullary and intramedullary implants. Intramedullary devices appear to have some advantages over extramedullary devices, which are biomechanically more stable under loading because their lever arm is short and their insertion is minimally invasive [2, 3]. Furthermore, immediate weight bearing is ensured, and postoperative morbidity remains low. Internal fixation with an intramedullary device has gained popularity for treatment of intertrochanteric femoral fractures. Despite good and reliable results, some typical failures and complications may occur [4, 5]. The most common complications are lag screw cut out from the femoral head and femoral fracture at the distal tip of the nail [6, 7], and complications related to the lag screw include medial pelvic migration [7] and lateral migration [8].

Here we present a rare complication of postoperative lag screw migration into the pelvis with no trauma. The patient was subsequently treated with lag screw removal and revision surgery with total hip arthroplasty.

## 2. Case Report

**Figure 1:**
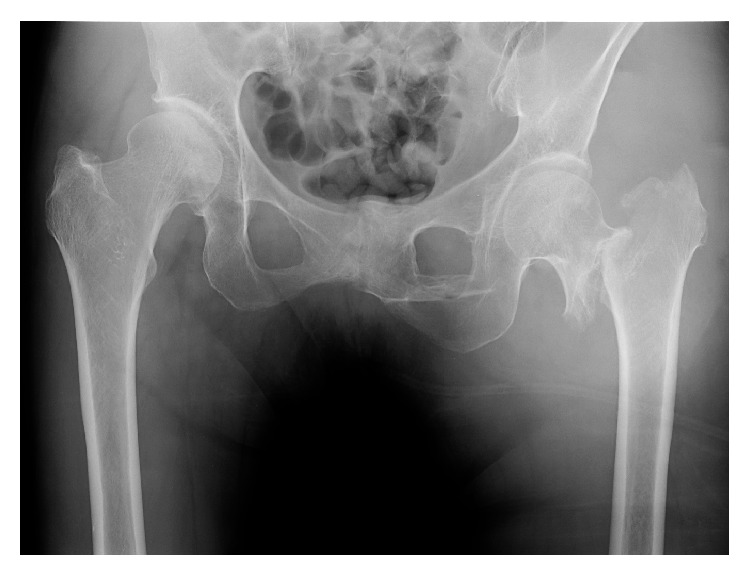
Radiograph showing a trochanteric fracture in the left femur, AO type 31-A2.2 on initial presentation.

**Figure 2:**
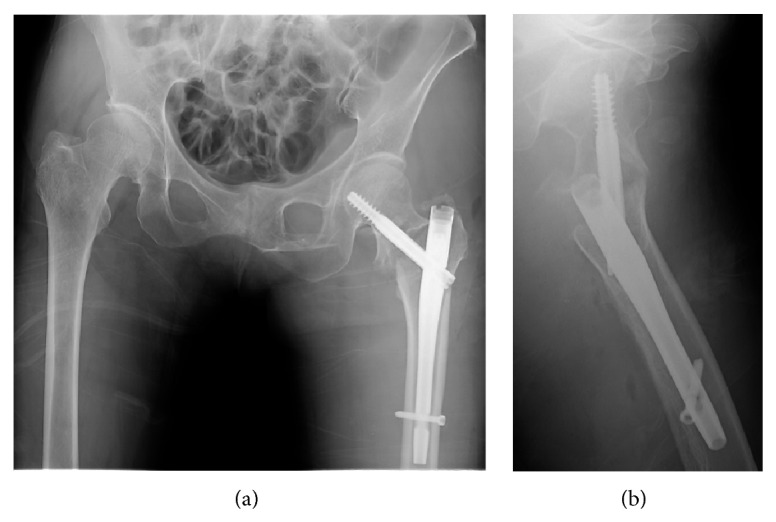
Radiographs after the osteosynthesis of the left trochanteric fracture. Anteroposterior (a) and lateral (b) views of the left femur showing insufficient reduction of the trochanteric fracture after implantation of a gamma 3 nail.

**Figure 3:**
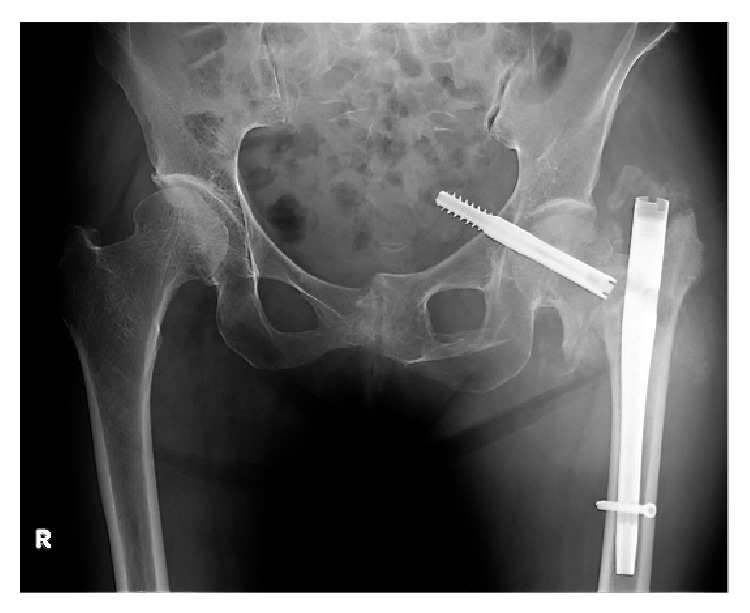
Radiograph showing redisplacement of the fracture and intrapelvic migration of the lag screw through the femoral head and the medial wall of the acetabulum.

**Figure 4:**
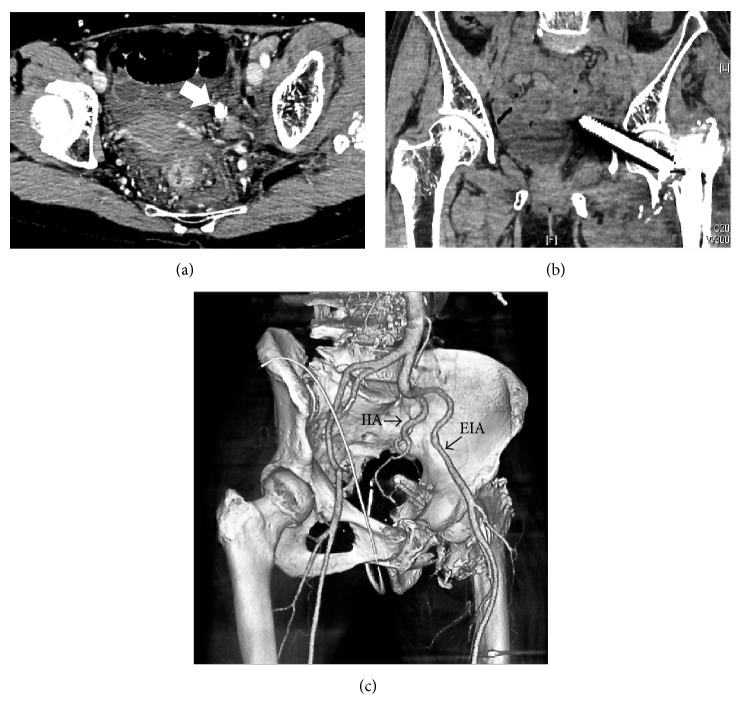
Contrast-enhanced computed tomography showing the lag screw located deep in the pelvis, between the internal and external iliac vessels, and tangent to the bladder and sigmoid. (a) Axial view (arrow: lag screw), (b) coronal view, and (c) reconstruction 3D image (EIA: external iliac artery, IIA: internal iliac artery).

**Figure 5:**
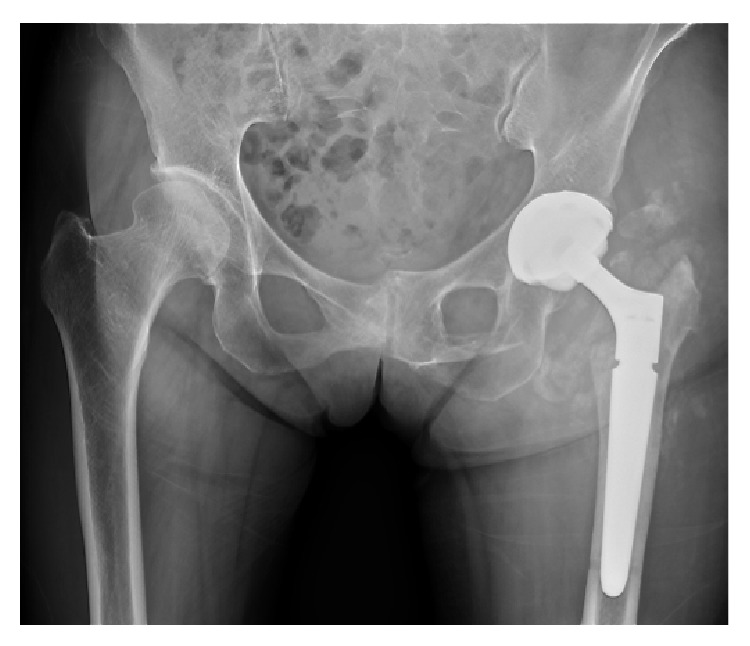
Postoperative radiographs of the left femur showing revision surgery with total hip arthroplasty.

A 63-year-old female was injured after falling off a bicycle and transported to a nearby emergency hospital by ambulance. On initial physical examination, tenderness and swelling were noted over the trochanteric region. She could not move her left leg actively because of severe left hip pain. She had no distal neurovascular deficits or other clinically apparent orthopedic or systemic injuries. Radiography revealed a trochanteric fracture in the left femur, AO type 31-A1.2 (Figure 1). She underwent closed reduction and internal fixation with a short gamma 3 nail (Stryker, Tokyo, Japan) according to the standard technique (Figure 2). The lag screw was inserted close to the subchondral bone, but eccentrically in the femoral head. No unusual events were noted during the operative period, and the early postoperative course was uneventful. The patient was mobilized and attained full weight-bearing status prior to discharge 1 month later. At 6 weeks after the first operation, she returned to the hospital with a 1-week history of progressive pain in her left hip, but no trauma. Radiography revealed redisplacement of the fracture and intrapelvic migration of the lag screw through the femoral head and the medial wall of the acetabulum, which was separated from the nail body (Figure 3). She was referred to our hospital for revision surgery. Intra-abdominal organ injuries were excluded on contrast-enhanced computed tomography (Figure 4). The lag screw was located deep in the pelvis, between the internal and external iliac vessels, and tangent to the bladder and the sigmoid, but no signs of pneumoperitoneum or hematuria were present. We scheduled a 2-stage operation. First, the migrated lag screw was removed without injury to other pelvic structures. Immediately after the surgery, the pelvis and abdomen were assessed by computed tomography, which revealed no intrapelvic damage due to removal of the migrated lag screw. In the second operation, 3 weeks after the removal surgery, cementless total hip arthroplasty with an autologous bone graft for the bony defect in the acetabulum due to lag screw penetration was performed (Figure 5).

The patient tolerated the procedure well and the postoperative period was uneventful. At the most recent followup 2 years after the revision surgery, she had fully recovered activities of daily living and did not require a walking aid.

## 3. Discussion

Postoperative medial lag screw migration into the pelvis is a rare complication. Although it has been termed “the Z effect,” a description more appropriate for 2-screw devices, the same phenomenon has also been noted in implants with a single femoral head fixation element, including the gamma nail [4, 7]. To our knowledge, only a few similar cases have been reported in the literature. Tauber and Resch [7] reported a case of sigmoid perforation after medial migration of a lag screw during gamma nailing but did not mention the cause of this complication. Rebuzzi et al. [9] reported that, out of 981 intramedullary hip fracture fixations, 21 had a “risky” screw position and, of them, 9 lag screws cut out and only 3 penetrated the acetabulum. This report showed that although medial migration is typically rare (<1%), it is relatively common when the screw position is deemed to be risky (43%). Commonly accepted operative predictors for fixation failure are quality of reduction, tip apex distance (TAD), and lag screw position within the femoral head and neck [10].

In our case, a short gamma 3 nail was placed, with the lag screw locked rotationally by the set screw, which controls not only rotation but also the excessive medial and lateral movement of the lag screw. The gamma nail is biomechanically constructed so that the lag screw slides in the lateral direction. One major advantage of this mechanism is controlled compression of the fracture with weight bearing, which helps in fracture healing [11, 12]. In the present case, the lag screw was not controlled in the medial direction and migrated into the pelvis through the femoral head and medial wall of the acetabulum. We asked the manufacturer to investigate the removed implant, but no manufacturing or structural defect in the implant was found. The migration was found to be the results of the set screw not being inserted appropriately to control the lag screw; the lag screw was not locked rotationally and thus could not prevent spinning of the femoral head. Furthermore, we assumed that insufficient fracture reduction and inadequate positioning of the lag screw promoted spinning of the femoral head. As a result, repeated axial loading in unstable fixation caused toggling of the nail within the femoral canal, leading to medial migration similar to what is seen in the Z effect. This is the same mechanism proposed by Weil et al. in their biomechanical study [6]. It is important to achieve anatomical reduction in order to place the lag screw in the optimal position. The placement of the lag screw close to the apex of the femoral head on A-P view with the central placement on the lateral view is essential for the outcome.

In summary, we report a rare case of postoperative lag screw migration into the pelvis treated with total hip arthroplasty. This case shows that optimal fracture reduction and positioning of the lag screw are the most important surgical steps in decreasing the risk of medial migration of the lag screw. Moreover, careful attention must be paid to subsequent steps such as set screw insertion to prevent complications.
